# Antihypertensive Drug Guanabenz Is Active *In Vivo* against both Yeast and Mammalian Prions

**DOI:** 10.1371/journal.pone.0001981

**Published:** 2008-04-23

**Authors:** Déborah Tribouillard-Tanvier, Vincent Béringue, Nathalie Desban, Fabienne Gug, Stéphane Bach, Cécile Voisset, Hervé Galons, Hubert Laude, Didier Vilette, Marc Blondel

**Affiliations:** 1 INSERM U613, Brest, France; 2 Univ Brest, Faculté de Médecine et des Sciences de la Santé, UMR-S613, Brest, France; 3 Etablissement Français du Sang (EFS) Bretagne, Brest, France; 4 CHU Brest, Hop Morvan, Laboratoire de Génétique Moléculaire, Brest, France; 5 CNRS UPS2682, Station Biologique, Protein Phosphorylation and Disease Laboratory, Place Georges Teissier, Roscoff, France; 6 Institut National de la Recherche Agronomique (INRA), UR892, Virologie Immunologie Moléculaires, Jouy-en-Josas, France; 7 INSERM U648, Laboratoire de Chimie Organique 2, Université Paris Descartes, Paris, France; The Scripps Research Institute, United States of America

## Abstract

**Background:**

Prion-based diseases are incurable transmissible neurodegenerative disorders affecting animals and humans.

**Methodology/Principal Findings:**

Here we report the discovery of the *in vivo* antiprion activity of Guanabenz (GA), an agonist of α2-adrenergic receptors routinely used in human medicine as an antihypertensive drug. We isolated GA in a screen for drugs active *in vivo* against two different yeast prions using a previously described yeast-based two steps assay. GA was then shown to promote ovine PrP^Sc^ clearance in a cell-based assay. These effects are very specific as evidenced by the lack of activity of some GA analogues that we generated. GA antiprion activity does not involve its agonist activity on α2-adrenergic receptors as other chemically close anti-hypertensive agents possessing related mechanism of action were found inactive against prions. Finally, GA showed activity in a transgenic mouse-based *in vivo* assay for ovine prion propagation, prolonging slightly but significantly the survival of treated animals.

**Conclusion/Significance:**

GA thus adds to the short list of compounds active *in vivo* in animal models for the treatment of prion-based diseases. Because it has been administrated for many years to treat hypertension on a daily basis, without major side-effects, our results suggest that it could be evaluated in human as a potential treatment for prion-based diseases.

## Introduction

Prion-based diseases are transmissible and invariably fatal neurodegenerative disorders for which no treatment is currently available [1]. Among these diseases are Creutzfeldt-Jakob disease (CJD) in humans, bovine spongiform encephalopathies in cattle and scrapie in sheep and goat. These diseases are associated with neuronal cell death which leads to characteristic “spongiform” vacuolation of the brain. According to the “protein-only” hypothesis, prions are solely composed of an abnormal form (PrP^Sc^) of the PrP protein (PrP^C^), a glycosyl-phosphatidyl inositol (GPI) anchored protein normally expressed at the surface of a number of cell types including in particular neurons. Transmissibility necessitates the conversion of host PrP^C^ by exogenous PrP^Sc^. PrP^Sc^ isoform displays a pronounced protease resistance, shows an increase in β-sheet structures and forms aggregates.

Based on the assumption that PrP^Sc^ corresponds to (or at least is part of) the pathogenic entity, various approaches aiming at reducing PrP^Sc^ or PrP^C^ levels for the development of prion disease therapies are currently explored (comprehensively reviewed in [1]). Indeed, recent results showed that depleting PrP^C^ from neurons of prion-infected mice in which the *Prnp* gene (encoding PrP protein) can be turned off, not only prevented progression of clinical disease, but also reversed spongiosis and early cognitive deficits and neurophysiological dysfunction [2], [3]. Thus approaches leading to reduction of endogenous PrP^C^ or PrP^Sc^ levels may well be effective after the appearance of symptoms. Among these approaches are passive or active immunisations [4], [5] whereas others aim at the identification of pharmacological compounds or of peptide aptamers [6] promoting PrP^Sc^ clearance. Some of these approaches are based on the use of cell-free systems [7]–[10] whereas others are based on the use of mammalian cells chronically infected with prions ([11]–[14], reviewed in [15]). In most of these assays, drugs efficiency is monitored by their effects on proteinase K resistant-PrP^Sc^ accumulation and sometimes by titration of infectivity levels. All these assays are time and money consuming, in particular because experiments have to be carried out in highly secured-laboratories. For this reason, we developed a rapid and economical budding yeast (*Saccharomyces cerevisiae*)-based two steps assay to screen for antiprion molecules [16], [17].

Indeed, since 1994 [18] it is known that budding yeast contains several proteins behaving like prions (reviewed in [19]) and several simple reporter systems have been developed to investigate their behavior. In the first step of our assay, molecules from various chemical libraries were isolated on the basis of their *in vivo* activity against the [*PSI* ^+^] yeast prion. In the second step, the active hits were tested against [URE3], a second yeast prion unrelated to [*PSI* ^+^]. Both steps are based on the use of a white and red colony coloration system: prion-containing yeast cells ([*PSI* ^+^] or [URE3] cells) form white colonies on rich medium (YPD) whereas cells in which the prion phenotype is cured ([*psi* ^−^] or [*ure3-0*] cells) grow as red colonies. Our initial assumption that prion-controlling mechanisms could be conserved from yeast to mammals was confirmed when most of the active compounds isolated in the yeast-based assay turned out to be also active to promote PrP^Sc^ clearance in three different mammalian cell-based assays [16], [20]. A number of chemical libraries have been screened using this method including the Prestwick Chemical Library®, a collection of compounds at least in phase II of clinical trials. This library is composed of 880 molecules, among which 90% are marketed drugs and 10% bioactive alkaloids or related substances, thus representing a high degree of drug-likeness. Screening of this drug library followed the “SOSA Approach” consisting of submitting to the screening target only a limited number of highly diverse drugs for which bioavailability and toxicity studies have already been performed and which have proven their usefulness in human [21]. The positive hits can then be used as starting points for drug optimization programs. However, if the initial hit(s) has sufficient affinity for the target, it can be immediately tested in patients. Here we report the identification, from this library, of *in vivo* activity of Guanabenz acetate against both yeast and mammalian prions.

## Results

### Guanabenz (GA) and Tacrine (TA) are active against yeast prions

**Figure 1:**
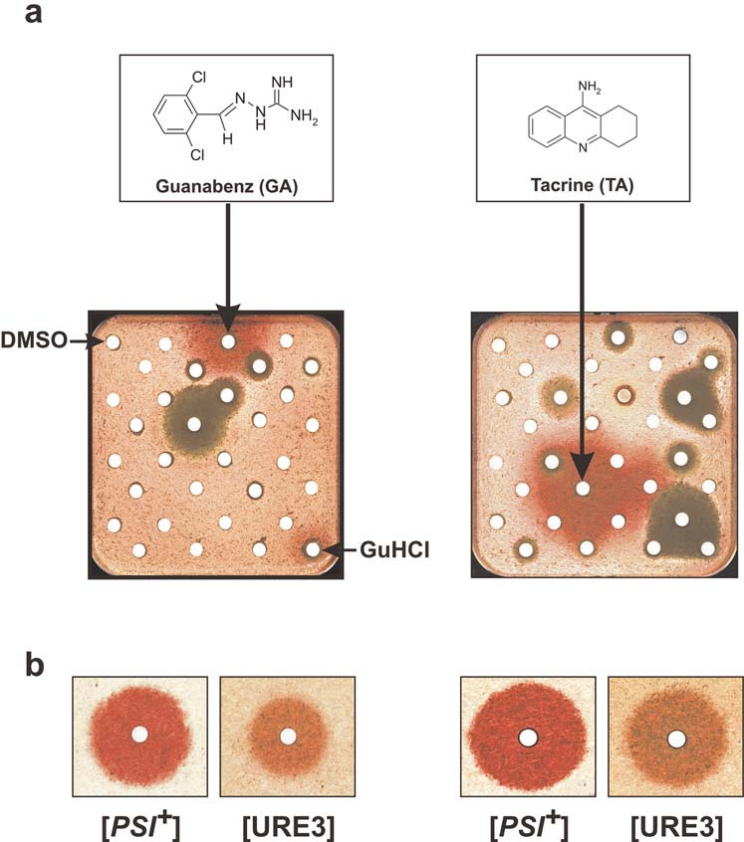
Guanabenz and Tacrine are active against yeast prions. a. An aliquot of an overnight culture a [*PSI+*] Strg6 strain (which grows as white colonies) was spread on Petri plates containing rich YPD (as indicated in the Materials and Methods section). Small filters (similar to the ones used for antibiograms) were then placed on the agar surface and individual compounds from the Prestwick chemical library® (5 µl of 2 mg/ml solutions) were applied to each filter, except for the top left filter where DMSO, the compounds vehicle was added (negative control) and for the bottom right filter where 5 µl of a 300 mM GuHCl solution in DMSO was added (positive control). The Petri plates were then incubated three days at 25°C. When a compound was active against [*PSI+*], a halo of red colonies appeared around the filter where it was spotted. Petri plates where Guanabenz (GA, left) and Tacrine (TA, right) were spotted are shown. The molecular structures of Guanabenz and of Tacrine are depicted on the top of the Petri plates. The red halos corresponding to GA- or TA-cured cells are indicated by arrows. Brown halos correspond to toxic compounds (see Materials and Methods). b. GA and TA were then tested against [URE3] prion using SB34 strain and the same kind of assay. As confirmations GA and TA were retested against [*PSI*+] prion. The same quantity (5 µl of a 5 mM solution) of GA (left) or TA (right) was applied on the filters thus allowing direct comparison of effect of both molecules on the two yeast prions.

Among others, the Prestwick Chemical Library® was screened using the yeast-based assay. As expected, we found Chlorpromazine (CPZ) and Quinacrine (QC), which are present in the Prestwick library, to be weakly active against yeast prions (**Figure S1**), as previously observed [16]. Eleven other compounds of the Prestwick Chemical Library® also showed weak effects (data not shown). Among all the other drugs, only two presented a strong activity against yeast prions: Tacrine (TA), a cholinesterase inhibitor in clinic for the symptomatic treatment of memory loss in Alzheimer's disease [22], and Guanabenz acetate (GA), an agonist of α2-adrenergic receptor used in the treatment of hypertension [23]. TA and GA were both active against [*PSI^+^*] prion (**Figure 1** panels **a** and **b**) and were then evaluated against the [URE3] prion and found to be also active (**Figure 1b**). Hydroxy Tacrine (hTA) was also found to be moderately active against [*PSI^+^*] prion (**Figure S2**, panel **a**).

### GA efficiently promotes ovine PrP^Sc^ clearance in an *ex vivo* cell-based assay

**Figure 2:**
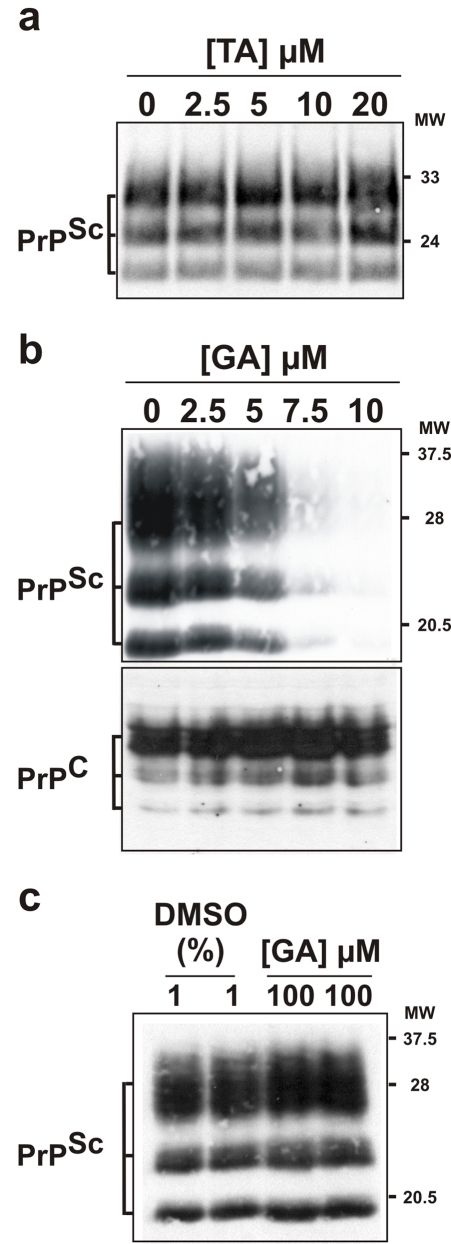
Guanabenz efficiently promotes ovine PrP^Sc^ clearance in an *ex vivo* cell-based assay. Scrapie-infected MovS6 cells were treated for six days with the indicated concentrations of TA (panel a) or GA (panel b, upper gel) and then lysed. Their effects on PrP^Sc^ accumulation were determined by Western blot analysis using an anti-PrP antibody. At the tested range of concentration, only GA was able to promote PrP^Sc^ clearance. The effect of GA on the steady-state level of PrP^C^ was determined in uninfected MovS6 cells (b lower gel). (c) Cell lysates of untreated scrapie-infected MovS6 cells were directly incubated with the indicated concentrations of GA or, as controls to the corresponding quantity of DMSO, the compound vehicle. PrP^Sc^ was then detected by Western blot analysis using an anti-PrP antibody. Molecular weights (MW, in kilodaltons) are indicated to the right of the blots.

The three compounds were then tested for their ability to promote PrP^Sc^ clearance in the mammalian MovS6 cell-based assay [14]. MovS6 cells correspond to a murine peripheral neuroglial cell line expressing ovine PrP gene (VRQ allele) under the control of its endogenous promoter. These cells are permissive to the 127S sheep scrapie agent [24]. Scrapie-infected MovS6 cells do not have any limitation in term of stability of infection (they remain stably infected even after long-term storage in liquid nitrogen) and accumulate high levels of both PrP^Sc^ and infectivity [14]. This assay was therefore chosen among other cell-based assays in order to test molecules in more stringent conditions and maybe avoid subsequent disappointing results *in vivo* [25], [26]. In addition and contrary to other cell-based assays, in the MovS6 cell-based assay cells already reached confluence when the drugs were added. A 6-days treatment with either TA or hTA in the 0 to 20 µM range of concentration did not prevent PrP^Sc^ accumulation in chronically-infected MovS6 cells as shown by Western blot analysis (**Figure 2**, panel **a** and **Figure S2**, panel **b**) suggesting that both molecules may be inactive against these mammalian prions in the tested range of concentration. In contrast, using the same conditions of treatment and a even lower range of concentration (0 to 10 µM), GA decreased PrP^Sc^ amount in the MovS6 cell cultures to levels barely detectable by Western blot (**Figure 2**, panel **b**, upper gel). By testing different concentrations, a dose-dependent antiprion effect was observed indicating that GA is active in the low micromolar range against PrP^Sc^ in the MovS6 cell-based system (**Figure 2**, panel **b**, upper gel). In the same set of experiments we followed by Western blot analysis the level of total PrP^C^ in non-infected MovS6 cells to determine if GA has any effect on the basal level of PrP expression in MovS6 cells, which could indirectly affect PrP^Sc^ accumulation (**Figure 2** panel **b**, bottom). As PrP level remained unchanged, we conclude that GA does not act by decreasing the steady state level of PrP.

### GA does not act directly on PrP^Sc^ aggregates

We next examined whether GA would be able to act directly on PrP^Sc^ aggregates, as previously described for other drugs such as Congo red or iododoxorubicin [27]–[29]. For this purpose, we incubated protein lysates from untreated infected MovS6 cells for five hours at 25°C without or with 100 µM of GA and then submitted them to proteinase K assay. As shown in **Figure 2c**, at 100 µM GA (which represents about 20 times the active concentration of GA in cell culture), the level of PrP^Sc^ did not change significantly meaning that this molecule does not exert any direct effect against PrP^Sc^ aggregates. In addition, GA was unable to inhibit the *in vitro* transconversion of PrP^C^ in PrP^Sc^ in a Protein Misfolded Cyclic Amplification assay (PMCA) and did not directly interact with PrP (Tribouillard-Tanvier *et al.* submitted), further confirming that this drug is probably not acting *in cis* on PrP.

### Activity of chemical derivatives of GA

**Figure 3:**
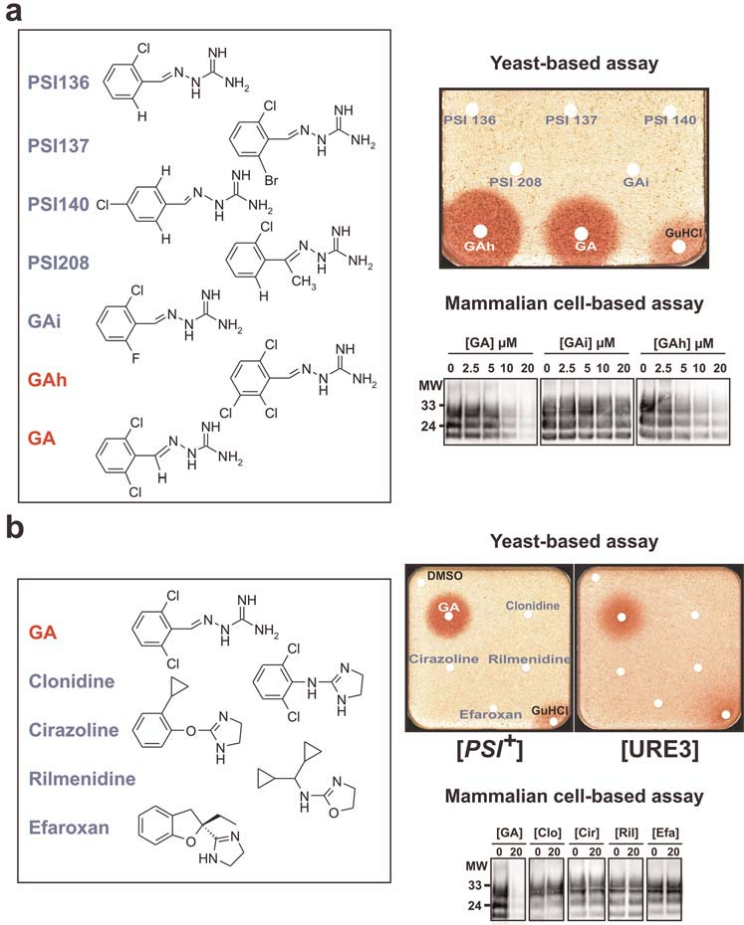
Activity of chemical derivatives of GA. a. The same quantity (5 µl of 5 mM solutions) of GA and of six chemical derivatives was spotted on filters as described in Figure 1. Note the lack of activity of GA analogs against [*PSI+*] yeast prion (upper right panel): for each of them, one of the two chlorines present in GA is missing (PSI 136, 137, 140, 208 and GAi). In contrast, adding one additional chlorine significantly increases the activity of the drug (GAh). GA, GAi and GAh were then tested for their ability to promote PrP^Sc^ clearance in the MovS6 cell-based system described above. Molecular weights (MW, in kilodaltons) are indicated on the right of the blots. b. Four other antihypertensive and/or known agonists or antagonists of α1- or α2-adrenergic receptors were tested against both [*PSI+*] and [URE3] yeast prions (top right) and mammalian prion using the MovS6 cell-based assay (bottom right).

Chemical derivatives of GA were then synthesized and tested against both yeast and mammalian prions using respectively the yeast-based and MovS6-based assays described above. As shown in **Figure 3a**, suppressing only one of the two chlorines (PSI 136) or even replacing it by fluorine (GAi) or by bromide (PSI 137) was sufficient to lead to a complete loss of activity in both assays. In contrast, adding supplementary chlorine onto GA (GAh) increased its activity against both [*PSI^+^*] and [URE3] and in the MovS6 cell-based assay, highlighting the importance of these two chlorines. This also confirmed the specificity of the screening method. Interestingly, activity of the various molecules in the MovS6 cell-based assay parallels their activity against yeast prions: GAi was also found inactive and GAh slightly more active than GA (bottom right panels).

### Antiprion activity of GA may not involve its agonist activity on α2-adrenergic receptors

To test if the antiprion activity of GA observed in MovS6 cells could be due to its agonist activity on α2-adrenergic receptors (which is responsible of its antihypertensive action), we tested the potential antiprion activity of Clonidine (CD), a compound which is pharmacologically and chemically very close to GA. Indeed CD is also an agonist of α2-adrenergic receptor used in clinic as an antihypertensive agent [30]. CD did not exhibit any antiprion activity in yeast nor in MovS6 cells (**Figure 3b**), suggesting that the antiprion activity of GA does probably not involve the same mechanism as the one linked to its antihypertensive action. The same result was obtained when using a variety of agonists or antagonists of α1- or α2-adrenergic receptors like Cirazoline (an agonist of α1- or α2-adrenergic receptors), Rilmenidine (an agonist of imidazoline I_1_ receptors) or Efaroxan (an antagonist of α2-adrenergic receptor) (**Figure 3b**). In addition, the antiprion activity of GA in *S. cerevisiae* cannot be due to its agonist activity on α2-adrenergic receptors since yeast does not contain adrenergic receptors. Taken together, these results suggest that the antiprion activity of GA does not use the cellular pathway leading to its antihypertensive effect.

### Activity of GA in a mouse model for prion-based disease

**Figure 4:**
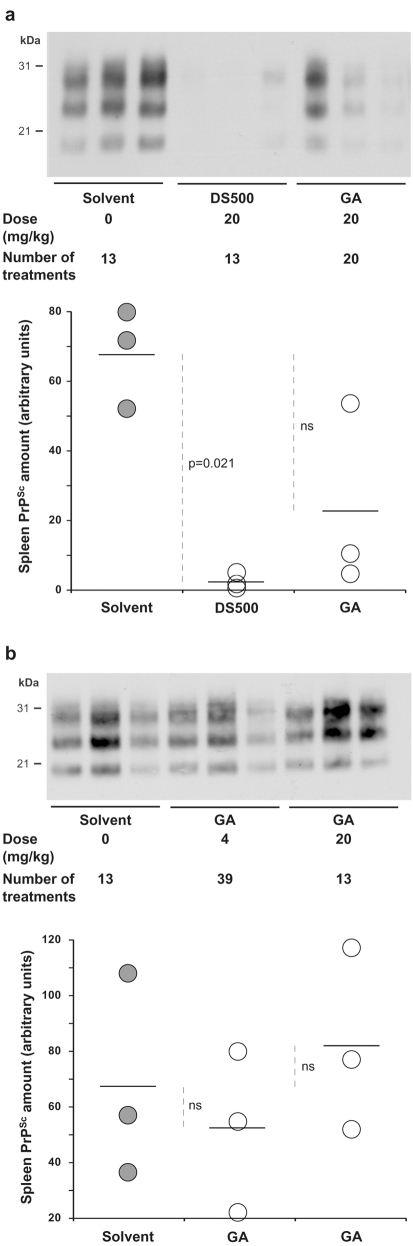
Effects of GA on PrP^Sc^ accumulation in scrapie-infected mouse spleens at mid-incubation. Transgenic mice overexpressing ovine PrP (tg338 line) were intraperitoneally infected with scrapie (127S strain) and treated with GA by the same route. Two independent experiments (shown respectively in panels a and b) have been performed, in which the dose and overall number of treatments varied, as indicated. Dextran Sulfate 500 (DS500, a) or GA solvent alone (5% glucose, a and b) served as positive and negative controls, respectively. In each treated group, triplicates of healthy mice were euthanized ∼50 days post-infection and the rate of PrP^Sc^ detectable in their spleens was determined by Western blot (see Materials and Methods). The same amount of spleen tissue material has been loaded on the gels. For each triplicate, Western blot of spleen PrP^Sc^ and densitometric quantification are shown. Each dot of the graph represents the PrP^Sc^ content of a single spleen. The dark horizontal line is the mean value. Statistical significance (*p*) was assessed using the non-parametric Kruskal-Wallis test. ns: not significant. Note that GA did not significantly affect spleen PrP^Sc^ level, although it showed a beneficial effect on mouse survival in both sets of independent experiments (see Table 1).

We finally evaluated the *in vivo* effect of GA on mammalian prion using a “rapid” mouse model for prion-based disease [29]. Tg338 mice overexpressing ovine PrP (VRQ allele) were intraperitoneally inoculated with a high dose of the 127S strain. We first examined whether repeated intraperitoneal injections of GA would be toxic for mice. Acute toxicity was observed over 40 mg/kg of GA. We thus chose not to exceed the dose of 20 mg/kg in subsequent treatments. Starting one day after infection (to avoid direct interaction between inoculum and the drug), tg338 mice were intraperitoneally treated with GA until the apparition of clinical signs in solvent-treated mice. GA has been tested in two independent sets of experiments at different doses and administration frequencies, as indicated. The effects of the molecule on the survival time of the mice and their statistical significance are summarized in **Table 1**. In both experiments, GA reproducibly induced a modest but statistically significant increase of the survival time of treated mice as compared to the solvent-treated mice (∼6%) when the drug was administered once or twice a week at 20 mg/kg. Increasing the number of treatments to three times a week, although at a lower dose (4 mg/kg) appeared more beneficial as survival time was increased by 19%, a percentage approaching that observed with dextran sulfate 500 (DS500; +26%), one of the best known anti-scrapie molecule because of its known inhibitory effects on scrapie primo-replication in the spleen of intraperitoneally-infected mice (see below and [29], [31]). Although the statistical significance of the difference observed between the two types of regimen is borderline (p = 0.06 Mann-Whitney U test), this result suggests that a more frequent treatment even with a lower dose could be *at minima* as efficient, in good agreement with the short half-life of GA *in vivo*, i.e. a couple of hours in the human body [23]. At terminal stage of the disease, GA has no significant effects on brain PrP^Sc^ accumulation as compared to solvent-treated mice (data not shown). In both experiments, triplicates of mice were also euthanized when still healthy at mid-infection (∼50 days) to assess GA effects on spleen PrP^Sc^ accumulation, a rapid method to identify drugs with any anti-prion potential [29]. In the first experiment, GA slightly inhibited PrP^res^ accumulation, although much less than DS500 (**Figure 4a**). However, quantification of the Western blot signals and comparison with solvent-treated animals failed to demonstrate any statistically significant effect of GA (**Figure 4a**). In the second experiment, no significant effects were observed, whatever the regimen administered (**Figure 4b**). Similar results were found at terminal stage of disease in this tissue (data not shown). This inconsistent inhibition of PrP^res^ accumulation in the spleen was reminiscent of that observed previously with MS-8209, an amphotericin B derivative [29] and suggests that GA does not act through inhibition of PrP^Sc^ accumulation in spleen.

**Table 1:** Effect of guanabenz acetate (GA) on the survival time of transgenic mice expressing ovine PrP (tg338 line) intraperitoneally inoculated with 127S scrapie strain.

	Treatment			(n/n_0_)^a^	Mean survival time	Delay	Significance *p* value^b^
	Number^c^	(mg/kg)	Frequency/week		(days±SEM)	(days)	(Mann-Whitney U test)
**1^st^ experiment**							
Solvent^d^	13	-	1	9/9	96±1		NA^e^
DS 500	13	20	1	10/10	121±3	25	<0.0001
GA	20	20	2 then 1^f^	9/9	102±2	6	0.022
**2^nd^ experiment**							
Solvent	13	-	1	6/6	95±1		NA
GA	13	20	1	9/9	101±1	6	0.0005
GA	39	4	3	10/10	113±11	18	0.0004

a diseased, PrP^Sc^ positive/inoculated mice.

b Comparison tested: drug treatment to solvent.

c Total number of treatments (see Methods).

d 5% glucose.

e NA: not applicable.

f 2× per week for 50 days then 1× per week.

Overall, we have shown in two independent experiments a significant effect of GA on the survival time of mice, in a model that might be difficult to cure, due to the high infectious load inoculated. We might anticipate that GA effects could be more pronounced in a less stringent model [32].

## Discussion

Taken together, our results indicate that GA, a drug already used in clinic for the treatment of hypertension [23], is active *in vivo* against both yeast and ovine prions. The data concerning the activity of GA against PrP^Sc^ were obtained in ovinized transgenic mouse and cell line models infected with sheep scrapie. It will be of interest to determine the potential of this molecule in other prions strains/species combinations, particularly humanized transgenic mice infected with Creutzfeldt-Jakob and variant Creutzfeldt-Jakob types [33]–[36]. Nevertheless, because GA does not seem to act directly on the PrP conversion process and because it is active against both yeast and ovine prions which are not related both in term of sequence and function, we believe this drug acts on common prion controlling mechanisms conserved in eukaryotes. Therefore our data suggest a potential new therapeutic indication for GA in the treatment of prion-based disease in mammals, including in humans. Indeed, GA has already been used safely in clinic for almost 25 years without any major side-effects and its pharmacology and toxicology are very well characterized. In addition, as an antihypertensive drug whose mechanism of action is an agonistic stimulatory effect of the central alpha-2 adrenergic receptors in the cardiovascular regulatory centers in the brainstem and spinal cord, GA is able to cross the blood brain barrier [37], [38]. We thus believe that GA could be evaluated in compassionate treatments for human prion-based disease. This is particularly appealing when one considers that quinacrine (QC) and chlorpromazine (CPZ), two compounds already in clinic for a long time for other applications and recently tested in human for compassionate treatment of CJD, were totally inefficient in human as well as in a mouse model similar to the one we used [25], [26], even in combination. Furthermore, these two molecules exhibit severe side-effects which contrast with GA [23], which has been safely used for years on a daily basis as an anti-hypertensive agent. Interestingly, both QC and CPZ are only weakly active in the yeast-based assay, which contrasts with GA. Moreover CPZ was not able to prevent PrP^Sc^ accumulation in primary cultures of tg338 mouse neurons infected with 127S scrapie strain [39]. It seems therefore reasonable to suggest that CPZ may be inefficient in prolonging the survival time of tg338 mice infected with this strain which is the one used in our study. The yeast-based assay could thus have a predictive value of the *in vivo* activity of antiprion drugs isolated in cell-based systems, especially since 6AP, another compound isolated as strongly active in the yeast based assay [16], exhibited some activity *in vivo* in the tg338 mouse model used in this study (VB and DV, unpublished data). Concerning TA, its inactivity in the MovS6 cell-based assay compared to the yeast-based system can have several explanations. Among them are trivial issues such as differences in cell permeability or in stability between the two systems. On the other hand it is well possible that TA targets a yeast cellular pathway that is either absent in mammals or that is too divergent to be efficiently targeted by this drug. Finally, GA also represents a good chemical scaffold to identify new potent antiprion molecules. The GAh derivative presented in this paper constitutes a good example. Compared to GA, these new compounds have not the advantage to be already in clinic but, on the other hand, they could have no effect on hypertension, a side-effect which could limit the use of GA as a treatment for prion based-diseases in human and animals. Therefore it will be important to determine the hypotensive and adrenergic receptor binding activities of GA derivatives for future use in anti-prion therapy.

## Materials and Methods

### Yeast strains and culture media

Yeast strains used in this study were as follows. Strg6: *Mata, erg6::TRP1, ade1-14, trp1-289, his3Δ200, ura3-52, leu2-3,112, [PSI+]* and SB34: *Mata, erg6::TRP1, dal5::ADE2, ade2-1, trp1-1, leu2-3,112, his3-11,15, ura2::HIS, [URE3]* and were grown as previously described [16], [17].

### Yeast-based antiprion screening assay

This assay was done as previously described [16], [17]. Briefly, yeast cells containing either [*PSI+*] or [URE3] prion lead to the formation of white colonies on rich (YPD) medium whereas, once cured of these prions ([*psi−*] or [ure3-0] cells), they lead to the formation of red colonies, due to the accumulation of a metabolic byproduct of the adenine biosynthesis pathway. An aliquot (350 µl of an 0.5 OD_600_ overnight culture) of [*PSI+*] or [URE3] cells (which grow as white colonies) were spread using sterile glass beads on square (12 cm×12 cm) Petri plates containing YPD medium supplemented with 200 µM Guanidine hydrochloride (-GuHCl- conditions where the sensitivity of the method is optimal). Sterile small filters (similar to the ones used for antibiograms) were then placed on the agar surface and individual compounds from the Prestwick chemical library® (5 µl of 2 mg/ml solutions) were applied to each filter, except for the top left filter where 5 µl of DMSO, the compounds vehicle was added (negative control) and for the bottom right filter where 5 µl of a 300 mM GuHCl solution in DMSO was added (positive control: GuHCl is a chemical inhibitor of Hsp104p, a protein chaperone essential for yeast prions propagation). The Petri plates were then incubated three days at 25°C. When a compound is active against [*PSI+*] or [URE3] prions, a halo of red colonies appear around the filter where it was spotted whereas colonies remain white in case of inactive compounds. The brown halos appearing around some of the filters are due to toxicity of the compounds deposited onto these filters which, by preventing yeast cell growth and therefore appearance of a lawn of yeast colonies (either as white or red colonies) reveals the brown/orange colour of the YPD medium. To confirm that potentially active compounds really cure yeast prions and do not act against the colorimetric system used as a reporter, cells from the red halos were streaked on a fresh drug-free YPD medium to control that they still form red colonies, an indication that [*PSI*+] or [URE3] prions were actually cured in these cells [17].

### PrP^Sc^ inhibition assay in MovS6 cells

Experiments were done as previously described [14]. Briefly, MovS6 cells chronically infected with 127S strain were treated for six days with the indicated concentrations of GA and then lysed. Cell lysates were then subjected to proteinase K digestion (only PrP^Sc^ is resistant to proteinase K) followed by Western blot analysis using the Sha31 anti-PrP antibody [40]. Western blot were analyzed by Enhanced Chemiluminescence (ECL, Amersham) using a Vilber-Lourmat Photodocumentation Chemistart 5000 imager which allow precise quantification of the signals.

### Effect of GA on the steady-state level of PrP^C^

Uninfected MovS6 cells were treated for six days with the indicated concentrations of GA and then lysed. Cell lysates were analyzed by Western blot using the Sha31 anti-PrP antibody [40] therefore allowing to determine potential effect of GA on the expression of ovine *Prnp* gene in MovS6 cells. Western blot were analyzed as mentioned above.

### Analysis of GA potential direct effect on PrP^Sc^ aggregates

Cell lysates of untreated scrapie-infected MovS6 cells (127S strain) were subjected directly to the indicated concentration of GA (final concentration) for 5 hours at 25°C or, as control, to the corresponding quantity of DMSO, the compound vehicle. They were then subjected to proteinase K digestion followed by Western blotting analysis using the Sha31 anti-PrP antibody [40]. Western blot were analyzed as mentioned above.

### Mouse model for prion-based disease

Experiments were done as previously described [41]. Mice overexpressing ovine PrP (tg338 line [24]) were infected intraperitoneally with 100 µl of the 127S scrapie strain at 0.02% (w/v) dose. This strain has an intracerebral infectious titre of ∼10^9^ ID_50_ U/g of brain [14]. Mice were then treated with GA the day following inoculation to exclude any direct interaction of the drug with the inoculum injected. The treatment was stopped around 85 days post-infection, a few days before the first symptoms appeared in the control mice group (mice treated with 5% glucose (solvent)) and the survival time in the different groups was determined. GA has been tested in two independent sets of experiments. In the first one, a 20 mg/kg dose of GA was intraperitoneally injected twice a week up to 50 days post-infection and then once a week, for a total of 20 treatments and a cumulative dose of 400 mg/kg. Its effects were compared to those of a 20 mg/kg dose of dextran sulphate 500 (DS500), intraperitoneally injected once a week (13 treatments, total dose 260 mg/kg). In the second set of experiments, GA was injected once a week at 20 mg/kg (13 treatments, total dose 260 mg/kg) or three times a week at 4 mg/kg (39 treatments, total dose 156 mg/kg). Mice were euthanized at terminal stage. Brains and spleens were collected and kept at −80°C for PrP^Sc^ analysis. In both experiments, some mice were also euthanized when still healthy in triplicates at 50 days post-inoculation to assess the effects of GA on spleen PrP^Sc^ accumulation. Brains and spleens tissues were homogenized at 20% (w/v) in 5% glucose with a Rybolyser (Hybaid). PrP^res^ was extracted according to the Biorad test protocol, by using 200 µg/ml proteinase K (Euromedex) for 10 min. at 37°C. After denaturation in Laemmli buffer, the samples were run on 12% NuPAGE gels (Invitrogen), electrotransferred onto nitrocellulose membranes and immunoblotted with 0.1 µg/ml anti-PrP antibody Sha31 [40]. The equivalent of 1 mg of spleen and 0.5 mg of brain tissue was loaded onto the gels. Immunoreactivity was visualized by chemiluminescence (GE Healthcare). The optical density of each sample (i.e. the three PrP^res^ glycoforms) was determined by the GeneTools software after acquisition of chemiluminescent signal with a GeneGnome digital imager (Syngene).

### Chemical compounds

Prestwick Chemical Library® was purchased from Prestwick Chemical company (Illkirch, France). It consists of a collection of 880 molecules, 90% being marketed drugs and 10% bioactive alkaloids or related substances, thus representing a high degree of drug-likeness. According to the manufacturer, the active compounds were selected for their high chemical and pharmacological diversity as well as for their known bioavailability and safety in humans. The compounds were supplied in 96-wells plates as 2 mg/ml solution in DMSO (approximatively 5 mM depending on the exact molecular weight (MW) of the considered compounds). GA, TA, Clonidine, Cirazoline, Rilmenidine, Efaroxan and GuHCl were purchased from Sigma.

### Synthesis of GA derivatives

Synthesis of all these compounds (GAh, GAi, PSI136, PSI137, PSI140 and PSI208) and of GA itself will be described elsewhere (FG, MB and HG, in preparation). Purity of all these molecules was determined using NMR, IR, mass spectrometry (MS) and HPLC and details can be given upon request.

## Supporting Information

Figure S1 Quinacrine and Chlorpromazine are only weakly active against yeast prions. a. An aliquot of an overnight culture of a [PSI+] Strg6 strain (which grows as white colonies) was spread on Petri plates containing rich YPD (as indicated in the Materials and Methods section). Small filters (similar to the ones used for antibiograms) were then placed on the agar surface and individual compounds from the Prestwick chemical library® (5 µl of 2 mg/ml solutions) were applied to each filter, except for the top left filter where DMSO, the compounds vehicle was added (negative control) and for the bottom right filter where 5 µl of a 300 mM GuHCl solution in DMSO was added (positive control). The Petri plates were then incubated three days at 25°C. When a compound was active against [PSI+], a halo of red colonies appeared around the filter where it was spotted. Petri plate where Quinacrine (QC, left) and Chlorpromazine (CPZ, right) were spotted is shown. The molecular structures of QC and of CPZ are depicted on the top of the Petri plate. The red halos corresponding to QC- or CPZ-cured cells are indicated by arrows. Brown halos correspond to toxic compounds (see Materials and Methods).
(2.51 MB TIF)

Figure S2 hydroxy Tacrine is moderately active against yeast prions and inactive to promote ovine PrPSc clearance in an ex vivo cell-based assay a. The same plate than in Figure 1a is shown and the position where hydroxy Tacrine (hTA) was loaded is indicated by an arrow. The molecular structure of hTA is depicted on the top right of the Petri plate. b. Scrapie-infected MovS6 cells were treated for six days with the indicated concentrations of hTA and then lysed. PrPSc levels were determined by Western blot analysis using an anti-PrP antibody. At the tested range of concentration, hTA, as TA, was unable to promote PrPSc clearance. Molecular weights (MW, in kilodaltons) are indicated to the right of the blot.
(2.83 MB TIF)
