# Supplementary figures and images for: Antihypertensive Drug Guanabenz Is Active In Vivo against both Yeast and Mammalian Prions
Source: PLoS One. 2008 Apr 23;3(4):e1981. doi: 10.1371/journal.pone.0001981 (PMC2291559; doi:10.1371/journal.pone.0001981)

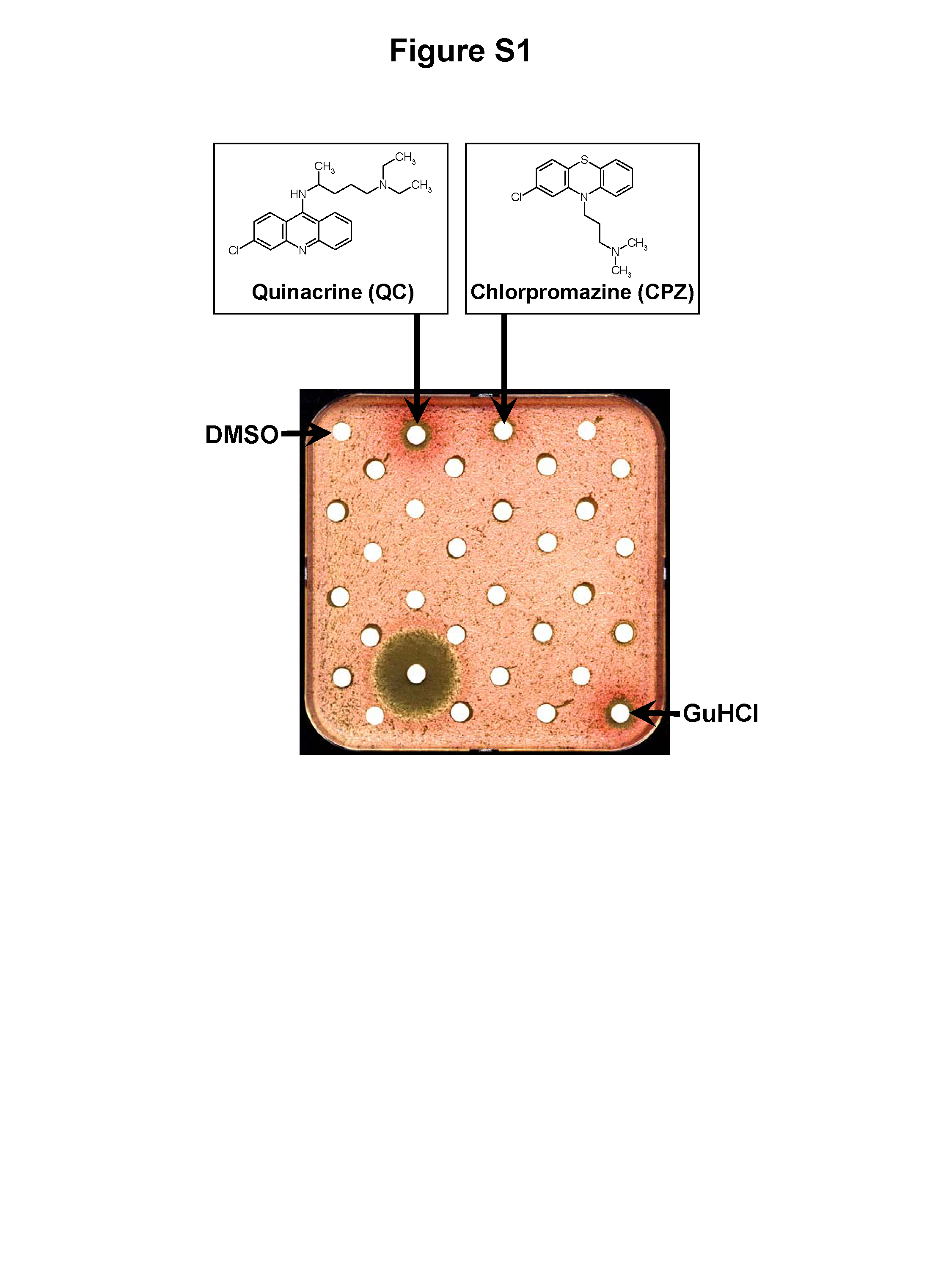

Supplement: Figure S1 — Quinacrine and Chlorpromazine are only weakly active against yeast prions. a. An aliquot of an overnight culture of a [PSI+] Strg6 strain (which grows as white colonies) was spread on Petri plates containing rich YPD (as indicated in the Materials and Methods section). Small filters (similar to the ones used for antibiograms) were then placed on the agar surface and individual compounds from the Prestwick chemical library® (5 µl of 2 mg/ml solutions) were applied to each filter, except for the top left filter where DMSO, the compounds vehicle was added (negative control) and for the bottom right filter where 5 µl of a 300 mM GuHCl solution in DMSO was added (positive control). The Petri plates were then incubated three days at 25°C. When a compound was active against [PSI+], a halo of red colonies appeared around the filter where it was spotted. Petri plate where Quinacrine (QC, left) and Chlorpromazine (CPZ, right) were spotted is shown. The molecular structures of QC and of CPZ are depicted on the top of the Petri plate. The red halos corresponding to QC- or CPZ-cured cells are indicated by arrows. Brown halos correspond to toxic compounds (see Materials and Methods). (2.51 MB TIF) [file pone.0001981.s001.tif]

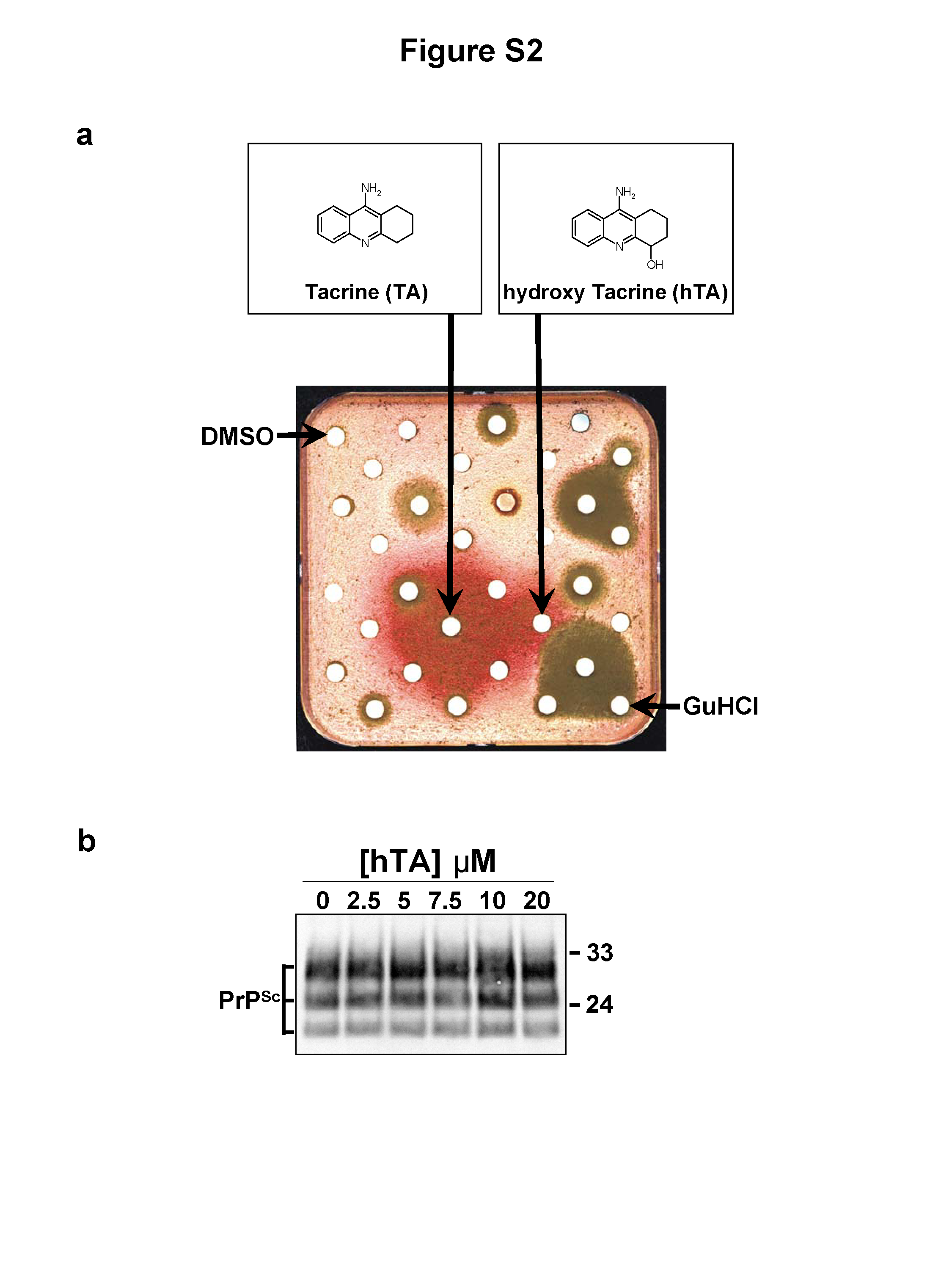

Supplement: Figure S2 — hydroxy Tacrine is moderately active against yeast prions and inactive to promote ovine PrPSc clearance in an ex vivo cell-based assay a. The same plate than in Figure 1a is shown and the position where hydroxy Tacrine (hTA) was loaded is indicated by an arrow. The molecular structure of hTA is depicted on the top right of the Petri plate. b. Scrapie-infected MovS6 cells were treated for six days with the indicated concentrations of hTA and then lysed. PrPSc levels were determined by Western blot analysis using an anti-PrP antibody. At the tested range of concentration, hTA, as TA, was unable to promote PrPSc clearance. Molecular weights (MW, in kilodaltons) are indicated to the right of the blot. (2.83 MB TIF) [file pone.0001981.s002.tif]
